# Improving Seismic Inversion Robustness via Deformed Jackson Gaussian

**DOI:** 10.3390/e23081081

**Published:** 2021-08-20

**Authors:** Suzane A. Silva, Sérgio Luiz E. F. da Silva, Renato F. de Souza, Andre A. Marinho, João M. de Araújo, Claudionor G. Bezerra

**Affiliations:** 1 Programa de Pós-Graduação em Ciência e Engenharia de Petróleo, Universidade Federal do Rio Grande do Norte, Natal 59078-970, RN, Brazil; Suzane.silva.065@ufrn.edu.br (S.A.S.); joaomedeiros@fisica.ufrn.br (J.M.d.A.); 2 Grupo de Imageamento e Inversão Sísmica, Universidade Federal Fluminense, Niterói 24210-346, RJ, Brazil; sergioluizsilva@id.uff.br; 3 Departamento de Física Teórica e Experimental, Universidade Federal do Rio Grande do Norte, Natal 59078-970, RN, Brazil; rf.desouza@dfi.uem.br (R.F.d.S.); andre.afonso@fisica.ufrn.br (A.A.M.); 4 Departamento de Física, Universidade Federal de Campina Grande, Campina Grande 58109-970, PB, Brazil

**Keywords:** Jackson statistics, inverse problems, robust inference, post-stack seismic inversion, deformed Gaussian distribution

## Abstract

The seismic data inversion from observations contaminated by spurious measures (outliers) remains a significant challenge for the industrial and scientific communities. This difficulty is due to slow processing work to mitigate the influence of the outliers. In this work, we introduce a robust formulation to mitigate the influence of spurious measurements in the seismic inversion process. In this regard, we put forth an outlier-resistant seismic inversion methodology for model estimation based on the deformed Jackson Gaussian distribution. To demonstrate the effectiveness of our proposal, we investigated a classic geophysical data-inverse problem in three different scenarios: (i) in the first one, we analyzed the sensitivity of the seismic inversion to incorrect seismic sources; (ii) in the second one, we considered a dataset polluted by Gaussian errors with different noise intensities; and (iii) in the last one we considered a dataset contaminated by many outliers. The results reveal that the deformed Jackson Gaussian outperforms the classical approach, which is based on the standard Gaussian distribution.

## 1. Introduction

Subsoil characterization is a topic of great economic and scientific interest, since many of its physical properties can be related to exploitable resources, such as hydrocarbons and gold [1]. In this regard, seismic inversion plays a fundamental role through the determination of quantitative models composed of physical properties that can not be directly observed [2]. Although direct measurements of subsurface physical properties are possible, they usually are costly, and coverage is much smaller than the area of interest. On the other hand, indirect measurements are cheaper and cover wider areas [3].

Inversion processes are required whenever the model’s parameters are inferred from indirect measurements. In the forward model, we calculate the output based on the input with a forward operator that maps each input to a single output [4]. In the inverse modeling, we try to map the output of the forward operator to its input. In ideal conditions, this would be obtained by the forward operator inversion, hence the name. However, in practice, this is not possible due to several factors that transform the operator inversion in an ill-posed problem [5]. For example, if we deal with a limited amount of data, there will be infinite models that generate the same output. The same problem occurs when the observed data posses any uncertainties.

There are many inversion methods which mitigate these problems. Here they will be classified into two groups: probabilistic and deterministic. Probabilistic methods try to retrieve all the possible solutions from the inversion problem and assign probability scores based on how likely they are. Consequently, this approach to the inversion results is an ensemble of parameters models. An example of such methods is the Bayesian inversion, which takes a prior parameter distribution, based on external knowledge, and infers the model parameters’ likelihoods based on the forward operator. On the other hand, deterministic methods return a single model for each set of data. The most common deterministic methods search the model parameters which minimize a so-called cost function of the discrepancies between the observed and modeled data (the residuals). An example of a deterministic method is the least square minimization, which minimizes the square of residuals.

There is a connection between the cost function minimization and probabilistic inversion based on the forward operators. The model obtained by minimizing a cost function is the most probable model of some likelihood function. Therefore, likelihood functions can be used to construct cost functions that present desirable properties. If the observations are formed in a way that the uncertainties are independent and identically distributed (*i.i.d.*), by a standard Gaussian likelihood [3], the maximum likelihood inversion will be equivalent to a least-squares residual minimization [4]. However, although complex systems that obey Gaussian statistics are quite common in nature, a wide variety of problems exhibit non-Gaussian behaviors, and therefore, the conventional approach which estimates physical parameters may result in biased model estimations [6]. In this way, non-Gaussian criteria are important for a more robust inversion process [7].

In order to mitigate the effect of non-Gaussian errors, several robust formulations have been proposed in the literature. Among them, we may mention the criteria based on heavy-tailed probability functions, such as Student’s *t* and Cauchy–Lorentz distributions [8,9]; hybrid functions [10,11,12]; and generalized probability distributions, such as the deformed Gaussian distributions in the context of Rényi [13,14,15], Tsallis [16,17,18,19], and Kaniadakis statistics [20,21,22]. Very recently, a connection between Jackson, Tsallis, and Hausdorff approaches in the context of generalized statistical mechanics was proposed [23]. Motivated by this, a question that naturally arises is: may a deformed Jackson $\bar{q}$-Gaussian be a better approach to mitigate the effect of non-Gaussian errors?

In this work, we consider a classic seismic inverse problem named post-stack inversion (PSI) [24], in which the main goal is the inference of the acoustic impedance (model parameters) from the stacked seismic signal [25]. We reformulate the PSI based on the assumption that the residuals are *i.i.d.* according to a deformed Jackson $\bar{q}$-Gaussian probability distribution [26] in the context of Jackson statistics [23]. The deformed Jackson $\bar{q}$-statistics are based on the one-parameter generalization of the exponential function known as deformed Jackson $\bar{q}$-exponential. In this way, we employ the $\bar{q}$-exponential function of the Jackson framework proposed in [27,28] to derive a robust cost function. In order to introduce the PSI based on the deformed Jackson $\bar{q}$-Gaussian distribution ($\bar{q}$-PSI), we organized the paper as follows. In Section 2, we formulate the $\bar{q}$-PSI from a probabilistic maximum-likelihood viewpoint, in which the classic PSI is a particular case. Section 3 is devoted to describe likelihood based on the deformed Jackson $\bar{q}$-statistics. In Section 4, we present the numerical simulations to test the robustness of our proposal in a realistic geological model widely used in geophysics. Finally, in Section 5, we discuss the advantages and disadvantages of the $\bar{q}$-PSI for overcoming outliers in measured data.

## 2. Post-Stack Seismic Inversion

In a seismic survey, a set of receivers are spread on the surface and along wellbores. A source emits a pulse which is reflected and then recorded by those receivers. This primary signal contains many undesirable features, such as secondary reflections. The seismic signal is then processed to mitigate some undesirable effects, enhance the principal reflection, and remove the secondary ones. After some data manipulations, migration, and stacking, we finally have the so-called post-stack signal, which consists of individual independent seismic traces $d_{j}^{\mathrm{obs}}\left(t\right)$, for each seismic receiver *j*. Here we will consider a 2D survey, for which j=0,1,⋯,L receivers are spread in a line, each one separated by a fixed distance Δx.

In the Robinson convolution model, the post-stack seismic trace $d_{j}\left(t\right)$ is related to the seismic reflectivity $r_{j}\left(t\right)$ by a convolution with a source wavelet w(t), which mathematically can be expressed as [24]

(1) $$d_{j}\left(t\right)=\frac{1}{2}\int_{-\infty }^{\infty }w\left(\tau \right)r_{j}\left(t-\tau \right)d\tau .$$

The seismic reflectivity can be calculated directly from the acoustic impedance $z_{i}\left(t\right)$ by using $r_{j}\left(t\right)=\left[z_{j}\left(t+\delta \tau \right)-z_{j}\left(t\right)\right]/\left[z_{j}\left(t+\delta \tau \right)+z_{j}\left(t\right)\right]$, where δτ is a small time step. A good approximation can be obtained from [25]

(2) $$r_{j}\left(t\right)=\frac{1}{2}\frac{d}{dt}\left\{\ln\left[z_{j}\left(t\right)\right]\right\}.$$

Since logarithm is an invertible function, we can invert $m_{j}\left(t\right)=\ln\;z_{j}\left(t\right)$ and recover the impedance by $z_{j}\left(t\right)=\exp\left[m_{j}\left(t\right)\right]$.

In practice, physical parameters are measured in discrete time intervals Δt; consequently, the observed signal is defined only for the times $d_{j}^{obs}\left(i\Delta t\right)=d_{ij}^{obs}$. Due to this limitation, we will consider only the discrete logarithmic seismic impedance, written as a matrix $m\in R^{n\times L}$. As a consequence, we can discretize the seismic impedance forward operator as

(3) d=WDm,

where d$\in R^{(n+m-2)\times L}$ is the modeled signal and $W\in R^{\left(n+m-2)\times (n-1\right)}$ is the wavelet convolution matrix defined as

(4) $$W=\left[\begin{array}{cccc} w_{1} & 0 & \cdots & 0\\ \vdots & w_{1} & \vdots & 0\\ w_{m} & \vdots & \ddots & 0\\ 0 & w_{m} & \vdots & s_{1}\\ \vdots & \vdots & \ddots & \vdots \\ 0 & \cdots & \cdots & w_{m} \end{array}\right].$$

Additionally, $\left\{w_{1},\cdots ,w_{m}\right\}\in R^{m}$ is the discretized seismic wavelet and $D\in R^{(n-1)\times n}$ is the first-order differential operator, given by

(5) $$D=\frac{1}{2}\left[\begin{array}{ccccc} -1 & 1 & 0 & \cdots & 0\\ 0 & -1 & 1 & \cdots & 0\\ \vdots & \vdots & \ddots & \ddots & 0\\ 0 & \cdots & \cdots & -1 & 1 \end{array}\right].$$

Finally, for the residuals $\Delta d\in R^{(n+m-2)\times L}$, the discrepancy between the modeled and observed data is expressed as $\Delta d=d^{obs}-d\left(m\right)$.

## 3. Likelihood Based on Jackson Statistics

In the classic approach, PSI assumes that the residuals r and d are independent and identically distributed (*i.i.d.*) by a standard Gaussian likelihood $L_{G}\left(m\right)$ expressed as [3]

(6) $$L_{G}\left(m\right)=\prod_{i,j}\frac{1}{\sqrt{2\pi }}\exp\left[-\frac{1}{2}{\left(d_{ij}^{obs}-d_{ij}\left(m\right)\right)}^{2}\right].$$

In the present work, we assume that the residuals obey a $\bar{q}$-Gaussian likelihood, based on the Jackson statistics, instead of the usual Gaussian distribution. In order to construct the $\bar{q}$-Gaussian, we consider Jackson’s $\bar{q}$-exponential [27,28,29]

(7) $$e_{\bar{q}}^{x}=\sum_{n=0}^{\infty }\frac{x^{n}}{{\left[n\right]}_{\bar{q}}!},$$

and the $\bar{q}$-analogue function, which satisfy the property exp(x)exp(−x)=1, so that $e_{\bar{q}}^{x}E_{\bar{q}}^{-x}=1$ [26]

(8) $$E_{\bar{q}}^{x}=\sum_{n=0}^{\infty }{\bar{q}}^{\frac{n(n-1)}{2}}\frac{x^{n}}{{\left[n\right]}_{\bar{q}}!}\;with\;e_{1}^{x}=\exp\left(x\right),$$

where $\bar{q}$ is a real number between 0 and 1 and ${\left[n\right]}_{\bar{q}}$ is the so-called $\bar{q}$-basic number defined as [27,28,29,30,31]

(9) $${\left[n\right]}_{\bar{q}}=\frac{{\bar{q}}^{n}-1}{\bar{q}-1}\;with\;{\left[n\right]}_{1}=n.$$

In this way, the $\bar{q}$-Gaussian distribution is defined as [26]

(10) $$p_{\bar{q}}\left(x\right)=e_{\bar{q}}^{-x}E_{\bar{q}}^{-x}=\frac{1}{Z_{\bar{q}}}\sum_{n=0}^{\infty }\frac{{\left(-1\right)}^{n}{\bar{q}}^{n(n+1)}}{{\left(1+\bar{q}\right)}^{n}{\left[n\right]}_{\bar{q}}!}x^{2n},$$

where $1/Z_{\bar{q}}$ is the normalization constant:

(11) $$Z_{\bar{q}}=2\sqrt{1-\bar{q}}\sum_{n=0}^{\infty }{\bar{q}}^{n}E_{{\bar{q}}^{2}}^{\frac{{\bar{q}}^{2(n+1)}}{\bar{q}-1}}.$$

**Figure 1:**
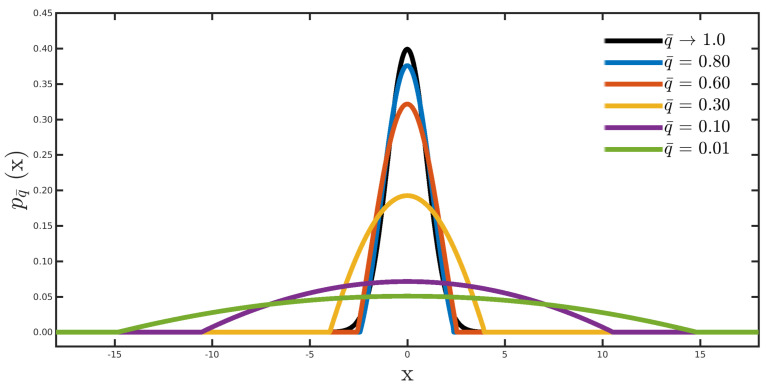
Probability plots of the $\bar{q}$-Gaussian distribution Equation (10) for typical $\bar{q}$-values, in which the black curve represents the standard Gaussian distribution ($\bar{q}\to 1.0$).

Figure 1 shows the graphical representation of the $\bar{q}$-Gaussian distribution Equation (10) for some $\bar{q}$-values, in which the standard Gaussian distribution ($\bar{q}\to 1.0$) is depicted by the black curve. We notice that the standard normal distribution gives high probability to residuals tightly distributed around zero (see the black curve in Figure 1). In contrast, our proposal residuals far away from zero receive increased probability as the parameter $\bar{q}$ decreases (see, for instance, the green curve in Figure 1), which is the characteristic of robust statistical estimators [6]. It is worth emphasizing that the $\bar{q}$-value indicates how deviated the behavior of the probabilistic error distribution is from Gaussian behavior, where $\bar{q}\to 1$ indicates that the inversion process assumes Gaussian errors, and 0<q<1 indicates that the errors do not obey Gaussian statistics, but rather a behavior far from a Gaussian distribution in the $\bar{q}\to 0$ limit-case.

Considering that the residuals are *i.i.d.* by Equation (10), the probability that a model m is the soil model is given by the likelihood $L_{\bar{q}}\left(m\right)$, expressed as

(12) $$\begin{array}{c} L_{\bar{q}}\left(m\right)=\prod_{i,j}p_{\bar{q}}\left(d_{ij}^{obs}-d_{ij}\left(m\right)\right). \end{array}$$

It is worth emphasizing that the most probable model can be recovered by searching the model which maximizes the likelihood. As mentioned before, instead of finding the maximum of the likelihood, we can construct a cost function whose minimum coincides with the likelihood maximum, and find this minimum instead. In this way, we can construct a function that is easier to work numerically, which is given by

(13) $$\begin{array}{cc} \phi_{\bar{q}}\left(m\right) & =-\ln\left[L_{\bar{q}}\left(m\right)\right]\\ \; & =-\sum_{i,j}\ln\left[\sum_{n=0}^{\infty }\frac{{\left(-1\right)}^{n}{\bar{q}}^{n(n+1)}}{{\left(1+\bar{q}\right)}^{n}{\left[n\right]}_{\bar{q}}!}{\left(d_{ij}^{obs}-d_{ij}\left(m\right)\right)}^{2n}\right]+\beta \left(\bar{q}\right), \end{array}$$

where $\beta (\bar{q})$ is a constant that depends only on $\bar{q}$. Since a logarithm is a monotonic function, its application does not shift the likelihood maxima and minima. Additionally, the logarithm is a bijective function which ensures that no minimum is fused or split. The maximum of likelihood $L_{\bar{q}}\left(m\right)$ is equal to the minimum of the cost function $\phi_{\bar{q}}\left(m\right)$. Therefore, the most probably model $\tilde{m}$ can be expressed as the following optimization problem:

(14) $$\tilde{m}=\underset{m}{argmin}\;\phi_{\bar{q}}\left(m\right).$$

We notice that in the $\bar{q}\to 1$ classic limit case, the $\bar{q}$-cost function, $\phi_{\bar{q}}$, retrieves the classic cost function, which is based on the Gaussian distribution.

## 4. Numerical Results

**Figure 2:**
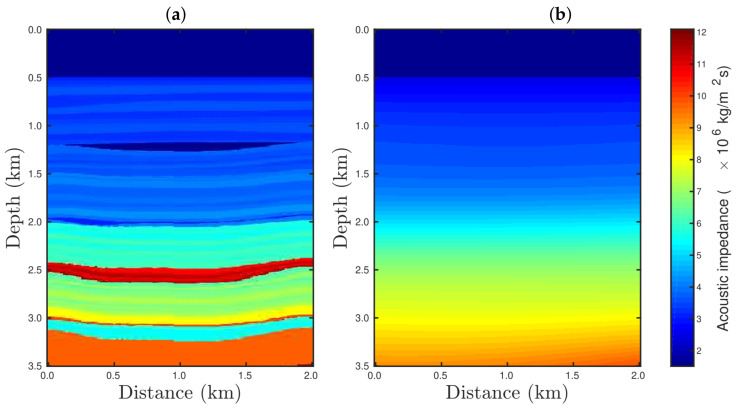
(**a**) The 2D Marmousi acoustic impedance model considered as the true model. (**b**) The initial model employed in the seismic inversion.

In order to demonstrate the robustness of our proposal, we consider a portion of the 2D Marmousi model [32,33]. Marmousi is a realistic geological model based on the Kwanza Basin (Angola) [34] which is largely used for testing new seismic imaging methodologies [35,36,37]. The area of study consists of water, gas, and oil sand channels, in addition to many reflectors and several geological strata, as depicted in Figure 2a. For the numerical simulations, we considered the seismic source to be a Ricker wavelet [38,39], with a peak frequency of 25 Hz, in all numerical experiments. The Ricker wavelet amplitude *w* according to time *t* is given by [38,39]

(15) $$w\left(t\right)=\left(1-2\pi^{2}\mu_{p}^{2}t^{2}\right)\exp\left(-\pi^{2}\mu_{p}^{2}t^{2}\right),$$

with $\mu_{p}$ being the peak frequency.

In order to perform the seismic inversion, we used the conjugate gradient (CG) algorithm [40,41], which iteratively updated the model’s parameters m according to

(16) $$m_{k+1}=m_{k}+\alpha_{k}h_{k}\left(m_{k}\right),$$

for $k=0,1,2,\cdots ,N_{iter}$. Here, $N_{iter}$ denotes the maximum number of iterations, α is the step-length, and h(m) is the so-called search direction [41]. In this work we considered the acoustic impedance model shown in Figure 2b as the initial model $m_{0}$. The step-length and the search direction at the *k*-th iteration were computed by [42,43]

(17) $$\alpha_{k}=\gamma \frac{\left|\right|m_{k}{\left|\right|}_{2}}{\left|\right|h_{k}{\left|\right|}_{2}}$$

and

(18) $$h_{k}=\left\{\begin{array}{cc} \;g_{0}\;, & if\;k=0\\ g_{k}+\zeta_{k}h_{k-1}, & for\;k=1,2,\cdots ,N_{iter}, \end{array}\right.$$

where γ is a scaling factor, $h_{k}=h_{k}\left(m_{k}\right)$, ${\left||.|\right|}_{2}$ is the Euclidean norm (or $l_{2}$-norm), $g=\nabla_{m}\phi_{Q}\left(m\right)$ is the gradient of the cost function, and ζ is given by [43]

(19) $$\zeta_{k}=\frac{g_{k}^{T}\left(g_{k}-g_{k-1}\right)}{g_{k-1}^{T}g_{k-1}}.$$

It is worth mentioning that in the numerical experiments, we set up $N_{iter}=30$ and γ=0.05, as suggested by [42].

### 4.1. Sensitivity to the Source Signature

**Figure 3:**
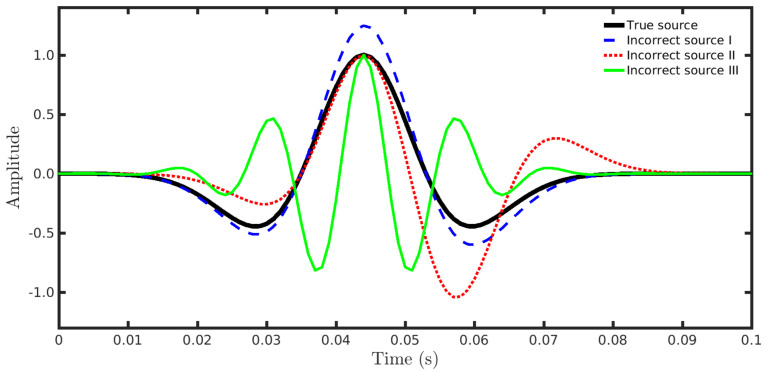
Source signatures used in this work. The solid black line represents the true seismic source (25 Hz Ricker wavelet). The dashed blue line, dotted red line, and solid green line represent the three incorrect source wavelets, respectively.

In practice, the source signature is unknown, and therefore, it is estimated from the seismic data, which introduces inaccuracies to the source wavelet. Consequently, inversion methods must be somewhat resilient when the forward modeled data are computed with an inaccurate seismic source. In this section, we present a numerical experiment for analyzing the sensitivity of our proposal to incorrect estimated sources. We used a noise-free dataset simulated by employing a 25 Hz Ricker wavelet as the true seismic signature (see the solid black curve in Figure 3).

In the seismic inversion process, we considered four distinct situations regarding the modeled seismic source. In the first one, we considered that the source signature was perfectly estimated, and in the last three, incorrect sources were used in the PSI. In particular, we considered the incorrect source signatures depicted in Figure 3. Incorrect source I (dashed blue line in Figure 3) was a modified version of the Ricker wavelet, as suggested in [44]; its amplitude $w_{1}\left(t\right)$ was associated with Equation (15) through the following relationship: $w_{1}\left(t\right)=w\left(t\right)exp\left(5t\right)$. Incorrect source II (dotted red line in Figure 3) was constructed from the derivative of incorrect source I, $w_{2}\left(t\right)\propto \frac{dw_{1}\left(t\right)}{dt}$, and incorrect source III (solid green line in Figure 3) was a real-valued Morlet wavelet: $w_{3}\left(t\right)=exp\left(-t^{2}/2\right)cos\left(5t\right)$.

**Figure 4:**
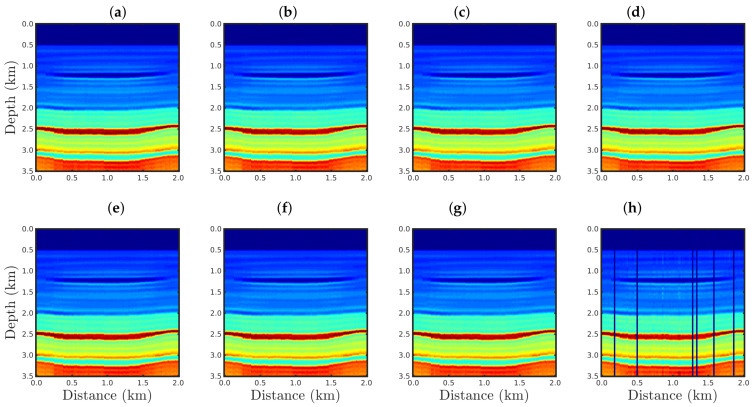
Models reconstructed for the correct source case using the PSI based on the (**a**) conventional approach ($\bar{q}\to 1$) or our proposal with: (**b**) $\bar{q}=0.9$, (**c**) $\bar{q}=0.6$, (**d**) $\bar{q}=0.4$, (**e**) $\bar{q}=0.3$, (**f**) $\bar{q}=0.1$, (**g**) $\bar{q}=0.01$, or (**h**) $\bar{q}=0.001$.

**Figure 5:**
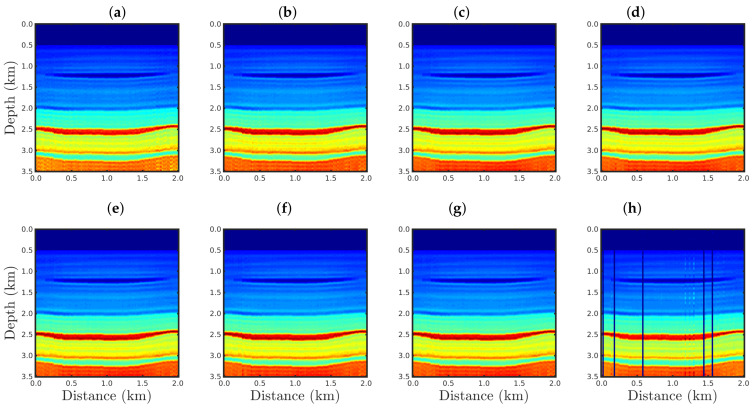
Models reconstructed for the incorrect source I case by using the PSI based on the (**a**) conventional approach ($\bar{q}\to 1$) or our proposal with: (**b**) $\bar{q}=0.9$, (**c**) $\bar{q}=0.6$, (**d**) $\bar{q}=0.4$, (**e**) $\bar{q}=0.3$, (**f**) $\bar{q}=0.1$, (**g**) $\bar{q}=0.01$, or (**h**) $\bar{q}=0.001$.

**Figure 6:**
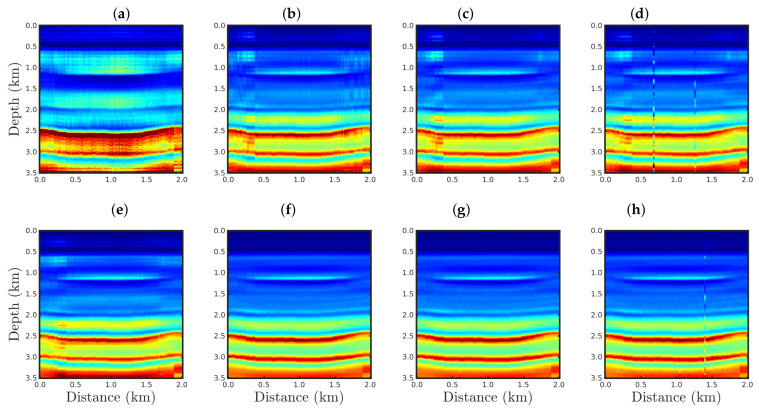
Models reconstructed for the incorrect source II case by using the PSI based on the (**a**) conventional approach ($\bar{q}\to 1$) or our proposal with: (**b**) $\bar{q}=0.9$, (**c**) $\bar{q}=0.6$, (**d**) $\bar{q}=0.4$, (**e**) $\bar{q}=0.3$, (**f**) $\bar{q}=0.1$, (**g**) $\bar{q}=0.01$, or (**h**) $\bar{q}=0.001$.

**Figure 7:**
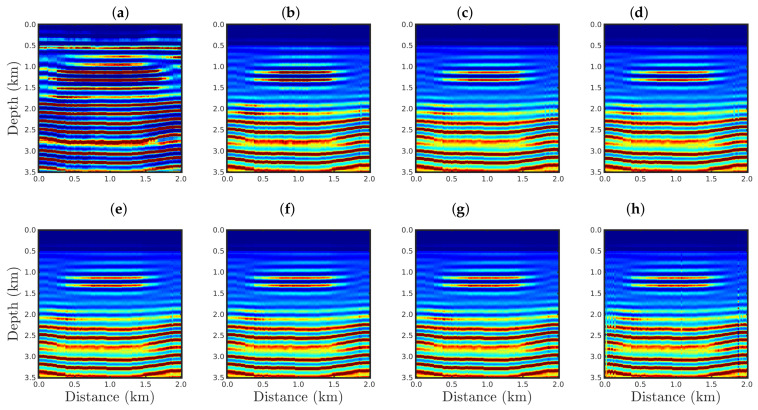
Models reconstructed for the incorrect source III case by using the PSI based on the (**a**) conventional approach ($\bar{q}\to 1$) or our proposal with: (**b**) $\bar{q}=0.9$, (**c**) $\bar{q}=0.6$, (**d**) $\bar{q}=0.4$, (**e**) $\bar{q}=0.3$, (**f**) $\bar{q}=0.1$, (**g**) $\bar{q}=0.01$, or (**h**) $\bar{q}=0.001$.

Figure 4, Figure 5, Figure 6 and Figure 7 show the PSI results for the four cases mentioned above. Through a visual inspection of Figure 4, we can see that if the modeled seismic source was correct, the PSI generated satisfactory results which are very close to the expected result (see Figure 2a), except in the $\bar{q}=0.001$ case, where some vertical artifacts can be observed in the reconstructed model (Figure 4h). Regarding the PSI results obtained using incorrect source I (Figure 5), we notice that the reconstructed models are similar to the case of the correct source (Figure 4). However, our proposal provided models with greater resolution than the conventional approach, especially for the $\bar{q}=0.01$ case (Figure 5g). In the case of incorrect source II, the conventional approach failed to reconstruct the acoustic impedance model (Figure 6a). In contrast, the accuracy of the PSI results based on our proposal is dependent on the $\bar{q}$-value. Indeed, as the $\bar{q}$-value decreased (which means a greater deviation from a Gaussian behavior), the reconstructed model became more reliable (see, for instance, panels (f) and (g) of Figure 6). Finally, if the modeled seismic source was too far from the ideal, which was the case in incorrect source III, both approaches failed completely, although our proposal generated models that resemble the true model, as depicted in Figure 7.

**Figure 8:**
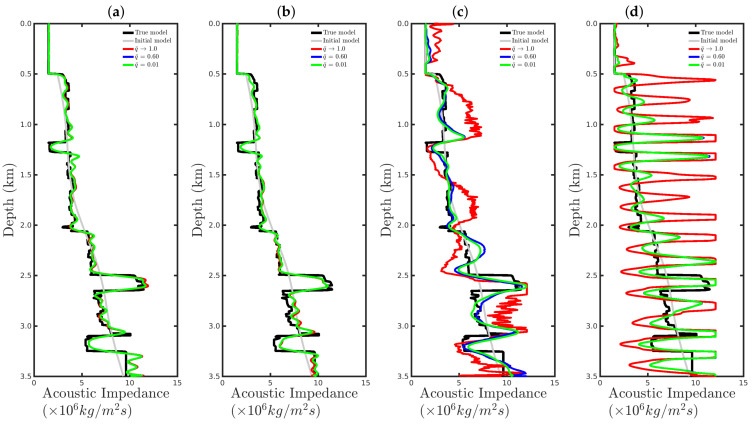
Vertical profiles of the true model (black curve), initial model (gray curve), and reconstructed models for the correct source in panel (**a**) and incorrect sources I, II, and III in panels (**b**–**d**), respectively. In this figure, we depict the central vertical well at Distance = 1.0 km.

Figure 8 shows a comparison between the vertical profiles of the central region (Distance=1 km) from the true model (black line), initial model (gray line), models reconstructed with the conventional approach (red line), and our proposal with $\bar{q}=0.60$ (blue line) and $\bar{q}=0.01$ (green line) for the four cases studied in this section. We notice that if the seismic source was correct or only a small disturbance was considered (incorrect source I), the resulting reconstructed acoustic impedance profiles were similar to the true profile (black line) regardless of the methodology used, as depicted in Figure 8a,b. However, if the source estimate was inaccurate, for instance, for incorrect source II, the conventional approach tended to fail (see the red line in Figure 8c). In contrast, our proposal was less sensitive to an error in the modeled seismic source (see blue and green lines in Figure 8c). Finally, if the modeled seismic signature had a very different shape from the true source, both approaches tended to fail.

**Figure 9:**
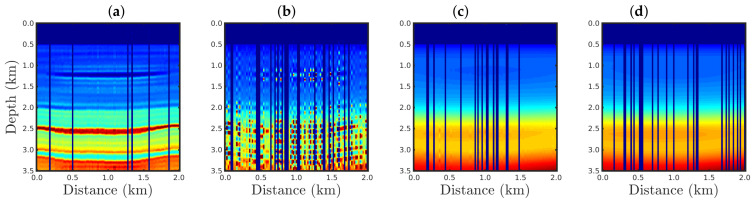
Model reconstructed for the correct source case by using the PSI based on our proposal with: (**a**) $\bar{q}=10^{-3}$, (**b**) $\bar{q}=10^{-4}$, (**c**) $\bar{q}=10^{-5}$, and (**d**) $\bar{q}=10^{-6}$.

Furthermore, it is important to notice the vertical fringes in the models reconstructed with our proposal for the $\bar{q}=0.001$ case, as shown in panel h of Figure 4, Figure 5, Figure 6 and Figure 7. This was due to the behavior of the $\bar{q}$-cost function (Equation (13)) in the $\bar{q}\to 0$ limit-case, which tends to be constant. In fact, in the $\bar{q}\to 0$ case, the optimization process was trapped in a local minimum, and the reconstructed models tended to assume the lower limit value of the model, generating blue fringes as depicted in Figure 9 for cases $\bar{q}=10^{-3},10^{-4},10^{-5}$, and $10^{-6}$.

### 4.2. Sensitivity to Gaussian Noise

**Figure 10:**
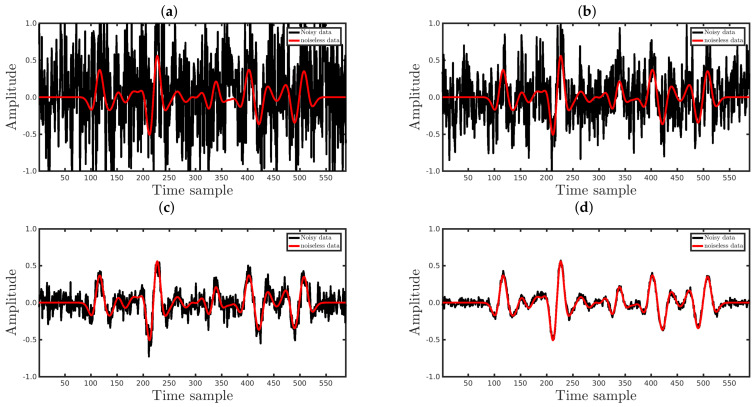
The black curves represent the observed data (waveforms) of the 51st seismic source (Distance=1km) contaminated by Gaussian noise with (**a**) SNR = 5 dB, (**b**) SNR = 10 dB, (**c**) SNR = 20 dB, and (**d**) SNR = 30 dB. The red curves represent the noiseless waveforms.

In this section, to demonstrate how the deformed Jackson Gaussian improves the seismic inversion robustness, we consider datasets polluted by Gaussian noise. In particular, we consider four noisy-circumstances regarding the signal-to-noise ratio (SNR): (i) SNR = 5 dB; (ii) SNR = 10 dB; (iii) SNR = 20 dB; and (iv) SNR = 30 dB. The SNR is defined as the ratio between the power of the noiseless waveform and the power of noise superimposed on the seismic data. In other words, the larger the SNR value, the smaller the Gaussian noise amplitude. Figure 10 shows an example of a waveform contaminated by Gaussian noise, with the different SNRs used in the present work being the black and red curves of the seismic data with and without noise, respectively.

**Figure 11:**
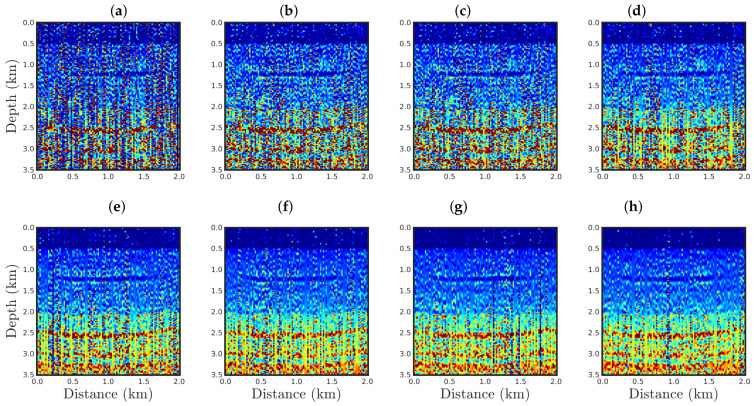
Models reconstructed for the Gaussian noise case, with SNR=5 dB, by using a PSI based on the (**a**) conventional approach ($\bar{q}\to 1$) or our proposal with: (**b**) $\bar{q}=0.9$, (**c**) $\bar{q}=0.75$, (**d**) $\bar{q}=0.6$, (e) $\bar{q}=0.4$, (**f**) $\bar{q}=0.3$, (**g**) $\bar{q}=0.1$, or (**h**) $\bar{q}=0.01$.

**Figure 12:**
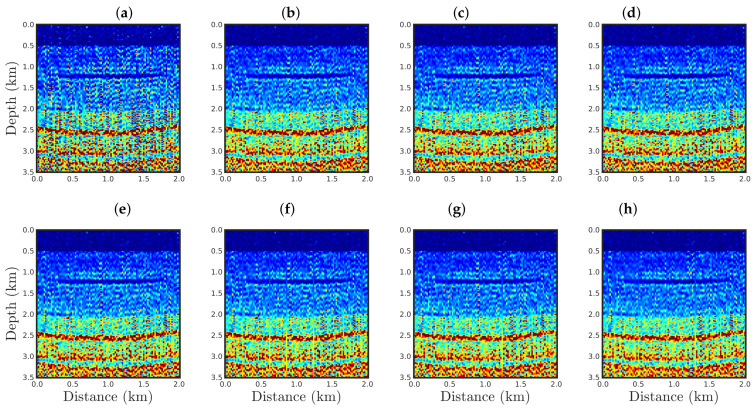
Model reconstructed for the Gaussian noise case, with SNR=10 dB, by using the PSI based on the (**a**) conventional approach (**$\bar{q}\to 1$**) or our proposal with: (**b**) $\bar{q}=0.9$, (**c**) $\bar{q}=0.75$, (**d**) $\bar{q}=0.6$, (**e**) $\bar{q}=0.4$, (**f**) $\bar{q}=0.3$, (**g**) $\bar{q}=0.1$, or (**h**) $\bar{q}=0.01$.

**Figure 13:**
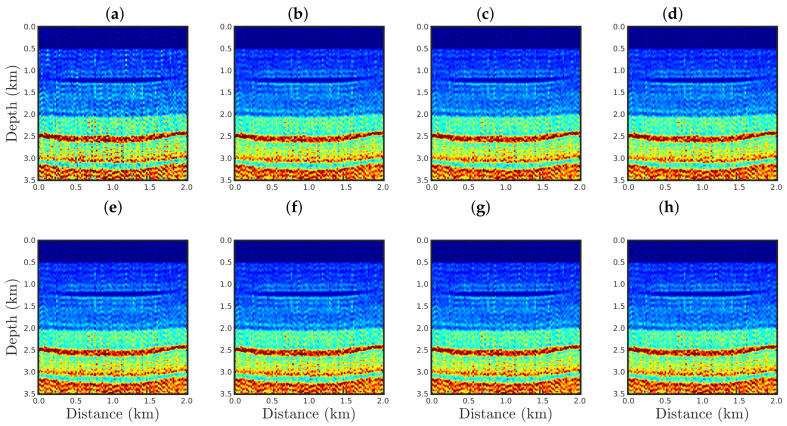
Model reconstructed for the Gaussian noise case, with SNR=20 dB, by using the PSI based on the (**a**) conventional approach (**$\bar{q}\to 1$**) or our proposal with: (**b**) $\bar{q}=0.9$, (**c**) $\bar{q}=0.75$, (**d**) $\bar{q}=0.6$, (**e**) $\bar{q}=0.4$, (**f**) $\bar{q}=0.3$, (**g**) $\bar{q}=0.1$, or (**h**) $\bar{q}=0.01$.

**Figure 14:**
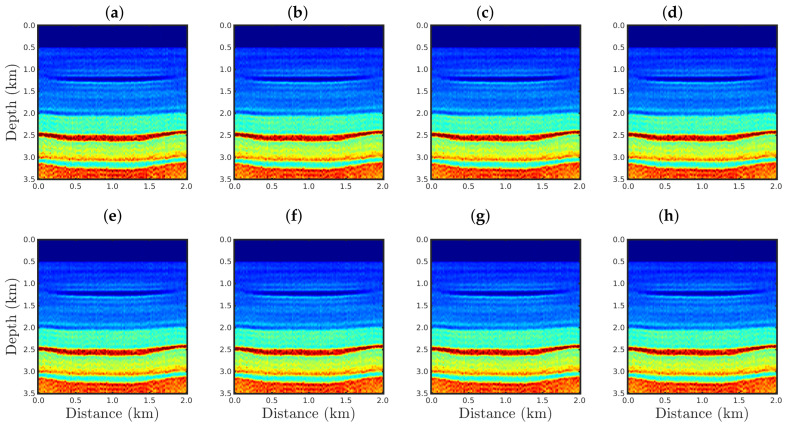
Model reconstructed for the Gaussian noise case, with SNR=30 dB, by using the PSI based on the (**a**) conventional approach (**$\bar{q}\to 1$**) or our proposal with: (**b**) $\bar{q}=0.9$, (**c**) $\bar{q}=0.75$, (**d**) $\bar{q}=0.6$, (**e**) $\bar{q}=0.4$, (**f**) $\bar{q}=0.3$, (**g**) $\bar{q}=0.1$, or (**h**) $\bar{q}=0.01$.

The PSI results using the conventional approach ($\bar{q}\to 1$) or our proposal with $\bar{q}=0.9$, 0.75, 0.6, 0.4, 0.3, 0.1, and 0.01 are illustrated in Figure 11, Figure 12, Figure 13 and Figure 14 for the cases SNR = 5, 10, 20, and 30 dB, respectively. From a visual inspection, it is remarkable that the reconstructed models are very similar. Indeed, the differences among the PSI results are not easily noticeable and can only be observed from error measures. Hence, we considered two measures to quantitatively compare the reconstructed models with the true model: first, we computed the normalized root-mean-square (NRMS):

(20) $$\mathrm{NRMS}={\left[\frac{\sum_{i=1}^{n}{\left(m_{i}^{true}-m_{i}^{inv}\right)}^{2}}{\sum_{i=1}^{n}{\left(m_{i}^{true}\right)}^{2}}\right]}^{1/2},$$

where $m^{true}$ corresponds to the true model and $m^{inv}$ is the inversion result. It is worth noting that an NRMS value close to zero means little error. The second measure used was the well-known Pearson’s coefficient (R) [45], which is a similarity measure. It is worth remembering thata Pearson’s coefficient score varies between 0 and 1, with being 0 a perfect “uncorrelation” and 1 being a perfect correlation between two samples. The measures for the four Gaussian noise scenarios are summarized in Table 1.

**Figure 15:**
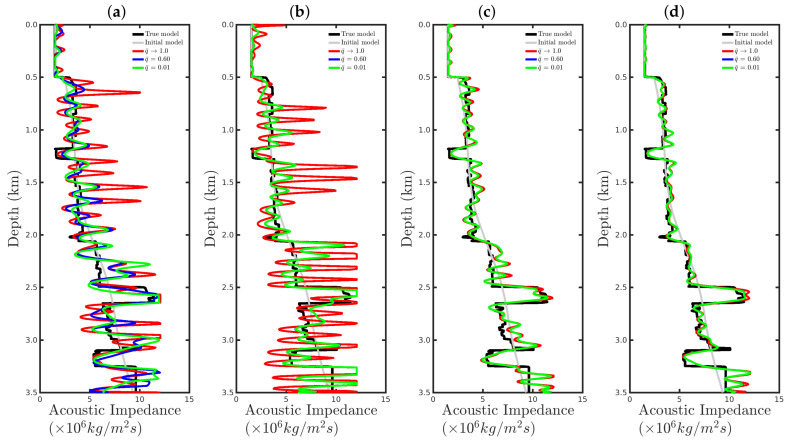
Vertical profiles of the true model (**black curve**), initial model (**gray curve**), and reconstructed models for the Gaussian noise case with (**a**) SNR = 5 dB, (**b**) SNR = 10 dB, (**c**) SNR = 20 dB, or (**d**) SNR = 30 dB. In this figure, we depict the central vertical well at Distance = 1.0 km.

**Figure 16:**
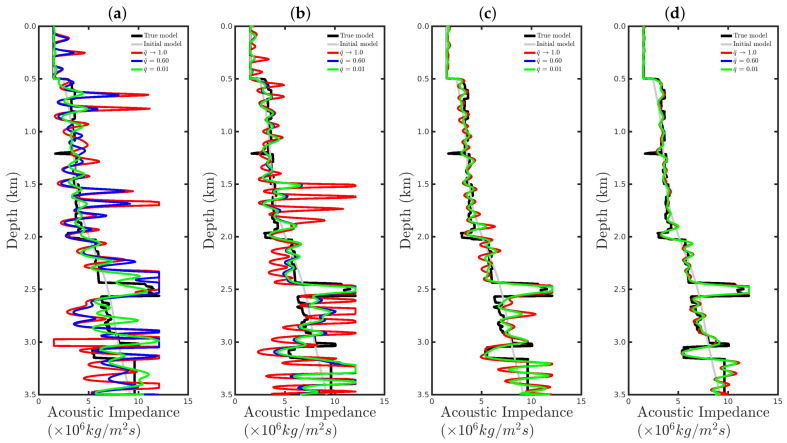
Vertical profiles of the true model (**black curve**), initial model (**gray curve**), and reconstructed models for the Gaussian noise case with (**a**) SNR = 5 dB, (**b**) SNR = 10 dB, (**c**) SNR = 20 dB, or (**d**) SNR = 30 dB. In this figure, we depict the central vertical well at Distance = 180 m.

Figure 15 and Figure 16 show comparisons between the vertical profiles of the central region (Distance=1 km) and a leftmost region (Distance=180 m) from the true model (black line), initial model (gray line), and models reconstructed with the conventional approach (red line) or our proposal with $\bar{q}=0.60$ (blue line) or $\bar{q}=0.01$ (green line) for the four Gaussian noise cases presented in this section. We notice that for a low SNR, for instance, in the SNR=5 dB and SNR=10 dB cases, the acoustic impedance profiles reconstructed by the conventional approach are very oscillatory (see red curves in panels (a) and (b) of Figure 15 and Figure 16) and do not match with the true model (black curves in Figure 15 and Figure 16). It is worth noting that the oscillatory behavior is also verified by the models resulting from our proposal. However, compared with the conventional approach, the profiles reconstructed by employing our proposal (see blue and green curves in panels (a) and (b) of Figure 15 and Figure 16) match those of the true model better. When the Gaussian noise was relatively weaker, that is, for the cases of high SNR, both approaches generated similar models (see panels (c) and (d) of Figure 15 and Figure 16), especially for the vertical profiles generated by our approach. The green and blue curves of those are superimposed in panels (c) and (d) of Figure 15 and Figure 16.

### 4.3. Sensitivity to Erratic Data (Outliers)

**Figure 17:**
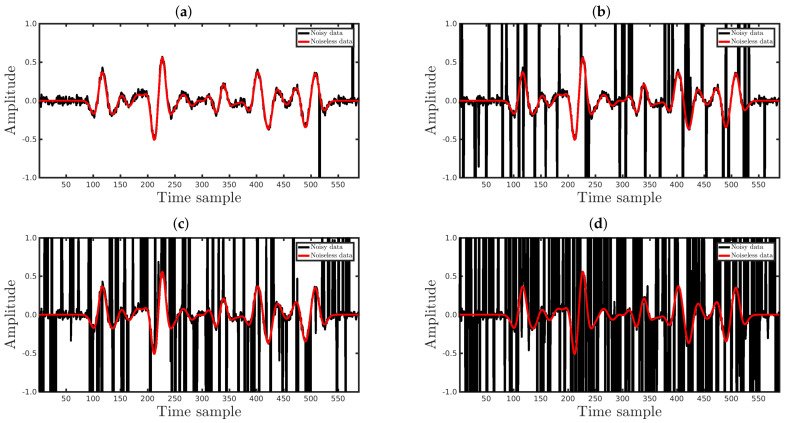
The black curves represent the observed data (**waveforms**) of the 51st seismic source (Distance = 1 km) contaminated by Gaussian noise and spiky-noise with (**a**) %Spike=0.5, (**b**) %Spike=10, (**c**) %Spike=20, or (**d**) %Spike=50. The red curves represent the noiseless waveforms.

**Figure 18:**
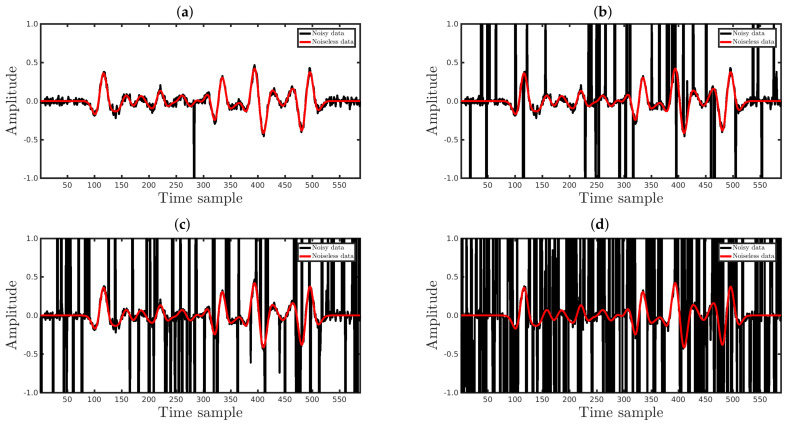
The black curves represent the observed data (**waveforms**) of the 10th seismic source (Distance = 180 m) contaminated by Gaussian noise and spiky-noise with (**a**) %Spike=0.5, (**b**) %Spike=10, (**c**) %Spike=20, or (**d**) %Spike=50. The red curves represent the noiseless waveforms.

Now, let us verify how the $\bar{q}$-cost function improves the PSI robustness in relation to non-Gaussian noise, which was represented in this work by erratic measures (outliers). We used seismic data contaminated by background noise and a set of outliers represented by spikes. In the simulations, the background noise consisted of Gaussian noise with a SNR of 30 dB, and the number of spikes was variable for each case. In the first case, we added spikes to 0.5% (%Spike=0.5) of the observed samples, randomly selected from a uniform distribution. For each selected point, the spike noise was calculated by making $d_{i}^{obs}={\left[d_{i}^{obs}\right]}_{Gaussian_{Noise}}+\alpha \times \beta$ with α∈[−2;2] and β, following a standard normal distribution. In the second case, 1.5% of the data were contaminated by spikes (%Spike=1.5), and so on, until %Spike=79.5 (%Spike=79.5), in regular intervals of 1%, totaling 80 different scenarios. For each scenario, we performed 100 inversions by considering the range $0.01\leq \bar{q}\leq 1$, totaling 8000 numerical experiments. Figure 17 and Figure 18 show examples of waveforms contaminated by Gaussian noise and spikes with the different %Spike values, in which the black and red curves represent the seismic data with and without spiky-noise, respectively.

**Figure 19:**
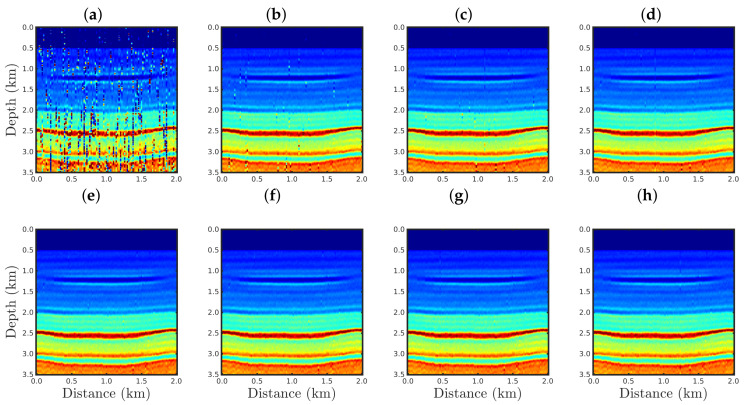
Model reconstructed for the %Spikes=0.5 case by using the PSI based on the (**a**) conventional approach (**$\bar{q}\to 1$**) or our proposal with: (**b**) $\bar{q}=0.90$, (**c**) $\bar{q}=0.75$, (**d**) $\bar{q}=0.60$, (**e**) $\bar{q}=0.40$, (**f**) $\bar{q}=0.30$, (**g**) $\bar{q}=0.10$, or (**h**) $\bar{q}=0.01$.

The conventional PSI result for the case %Spike=0.5 is shown in Figure 19a. As expected, a handful of outliers was sufficient to render the conventional approach inappropriate, as seen through the many artifacts in the estimated model. In contrast, our proposal was efficient to mitigate the effects of the outliers regardless of the *q*-values used, as depicted in Figure 19b–h.

**Figure 20:**
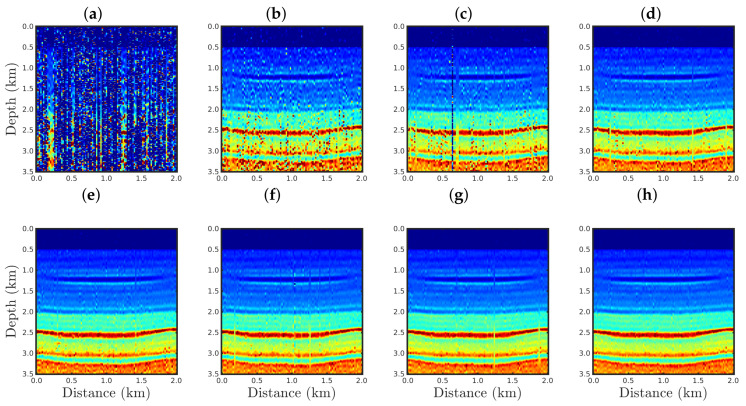
Model reconstructed for the %Spikes=10 case by using the PSI based on the (**a**) conventional approach (**$\bar{q}\to 1$**) or our proposal with: (**b**) $\bar{q}=0.90$, (**c**) $\bar{q}=0.75$, (**d**) $\bar{q}=0.60$, (**e**) $\bar{q}=0.40$, (**f**) $\bar{q}=0.30$, (**g**) $\bar{q}=0.10$, or (**h**) $\bar{q}=0.01$.

**Figure 21:**
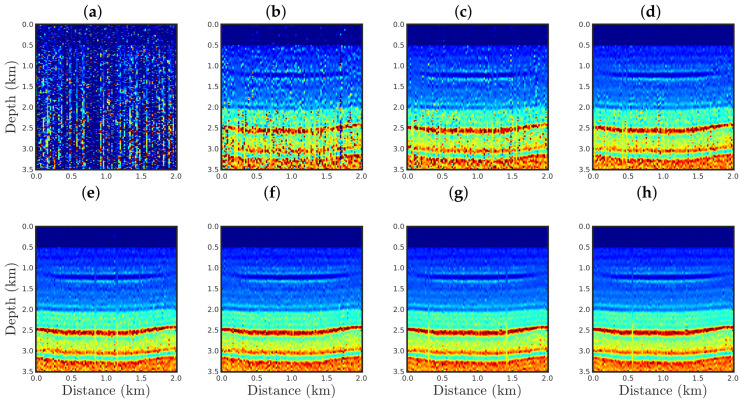
Model reconstructed for the %Spikes=20 case by using the PSI based on the (**a**) conventional approach (**$\bar{q}\to 1$**) or our proposal with: (**b**) $\bar{q}=0.90$, (**c**) $\bar{q}=0.75$, (**d**) $\bar{q}=0.60$, (**e**) $\bar{q}=0.40$, (**f**) $\bar{q}=0.30$, (**g**) $\bar{q}=0.10$, or (**h**) $\bar{q}=0.01$.

**Figure 22:**
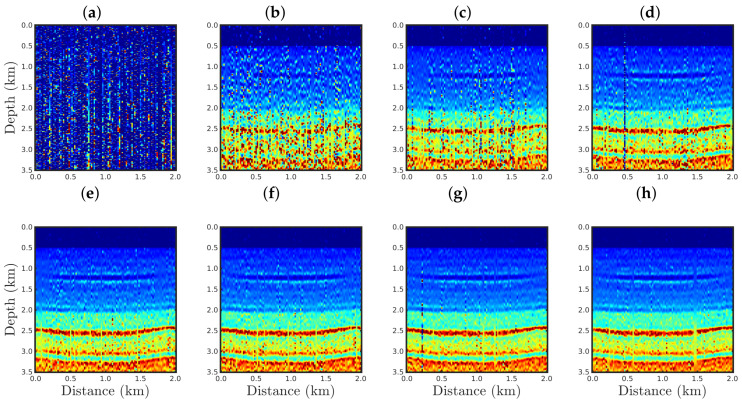
Model reconstructed for the %Spikes=50 case by using the PSI based on the (**a**) conventional approach (**$\bar{q}\to 1$**) or our proposal with: (**b**) $\bar{q}=0.90$, (**c**) $\bar{q}=0.75$, (**d**) $\bar{q}=0.60$, (**e**) $\bar{q}=0.40$, (**f**) $\bar{q}=0.30$, (**g**) $\bar{q}=0.10$, or (**h**) $\bar{q}=0.01$.

In the cases in which the seismic dataset was heavily contaminated by spikes, the conventional approach totally failed, as illustrated in panel a of Figure 20, Figure 21 and Figure 22 for the cases %Spike=10, 20, and 50, respectively. Indeed, conventional PSI was unable to identify any geological strata associated with the acoustic impedance contrast present in the true model (see Figure 2a). In this regard, we noticed that the conventional framework deteriorated the reconstructed models in comparison to the initial model (see Figure 2b). Regarding the $\bar{q}$-PSI, we noticed that even when the observation was highly-contaminated by spikes, the reconstructed models exhibited the main geological features of the true model, especially as the $\bar{q}$-value decreased, as depicted in panels (b)–(h) of Figure 20, Figure 21 and Figure 22.

**Figure 23:**
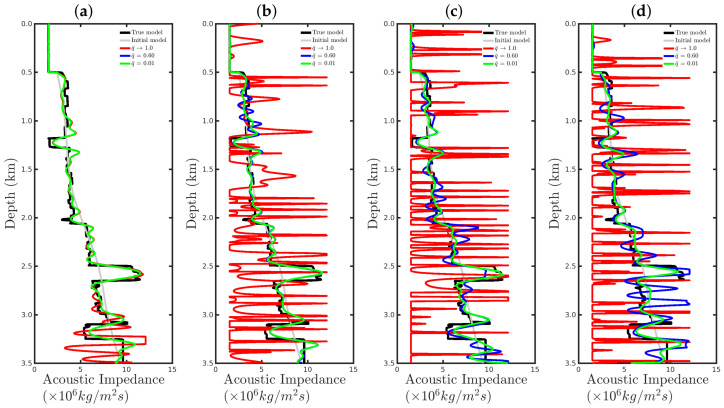
Vertical profiles of the true model (**black curve**), initial model (**gray curve**), and reconstructed models for the spiky-noise cases with (**a**) %Spikes=0.5, (**b**) %Spikes=10, (**c**) %Spikes=20, and (**d**) %Spikes=50. In this figure, we depict the central vertical well at Distance = 1.0 km.

**Figure 24:**
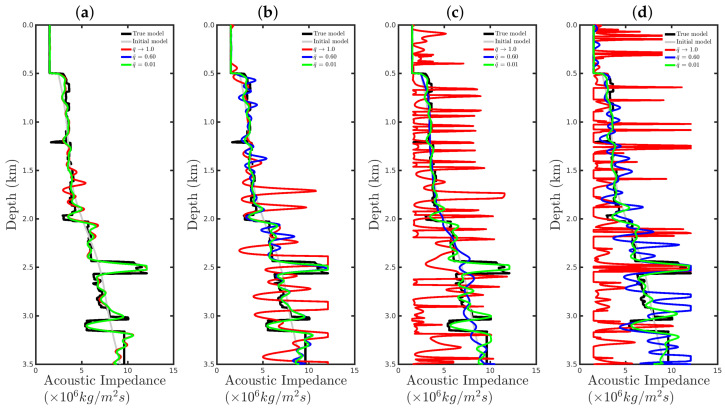
Vertical profiles of the true model (**black curve**), initial model (**gray curve**), and reconstructed models for the spiky-noise cases with (**a**) %Spikes=0.5, (**b**) %Spikes=10, (**c**) %Spikes=20, and (**d**) %Spikes=50. In this figure, we depict the central vertical well at Distance = 180 m.

Figure 23 and Figure 24 show comparisons between the vertical profiles of the central region (Distance=1 km) and a leftmost region (Distance=180 m) from the true model (black line); initial model (gray line); and models reconstructed with the conventional approach (red line) and our proposal with $\bar{q}=0.60$ (blue line) or $\bar{q}=0.01$ (green line) for the %Spike=10, 20, and 50 cases. In these figures, the red curves refer to the acoustic impedance profiles estimated using the conventional approach, in which the dissimilarity with the true model (black curve) is remarkable. In contrast, our proposal proved to be efficient at mitigating the effects of the outliers in the seismic dataset, especially for the $\bar{q}=0.1$ case, as depicted by the green curves in Figure 23 and Figure 24.

**Figure 25:**
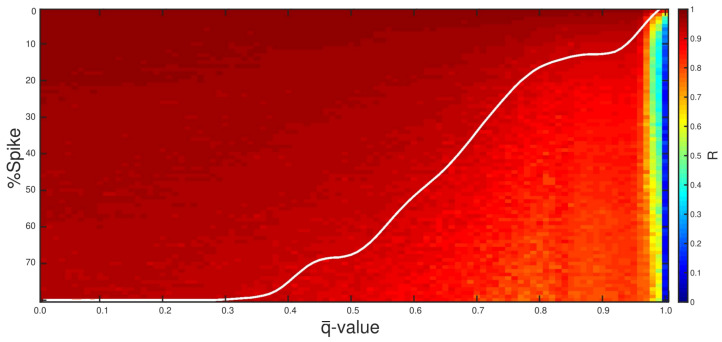
A heatmap as a graphical representation of Pearson’s *R* for the numerical experiments carried out in this work, in which the white curve indicates the R = 0.8 case.

From a quantitative viewpoint, let us analyze the quality of the $\bar{q}$-PSI results. First, we compute the correlation strength *R* between the reconstructed models and the true model through Pearson’s *R* correlation coefficient [45]. The Pearson’s R values for the numerical experiments are summarized in the heatmap shown in Figure 25, where the red colors indicate greater correlation between the reconstructed and the true model, and the blue indicates minor correlation. As a visual guide, we also added a white curve separating the heatmap into two regions, the upper region for which R≤0.8 and the lower region for which R>0.8. We notice that as the number of spikes increases, the best results are associated with the lowest $\bar{q}$-values. Since spikes are non-Gaussian errors, as the amount of spikes is increased, the deviation of the Gaussian behavior is also increased, which hinders the parameter recovery with the conventional approach. Even though a better performance of the $\bar{q}$-PSI inversion could be expected, it still impressive that high values of *R* were obtained even with a large amount of spike noise such as 79.5%.

## 5. Final Remarks

In this work, we introduced a new cost function to mitigate spurious measurements’ influences on the seismic inversion process. In particular, we considered a classical geophysical inverse problem named post-stack inversion (PSI), for which we applied the approach proposed here, the so-called $\bar{q}$-PSI, which uses deformed Jackson $\bar{q}$-statistics. With a realistic geological model, the numerical experiments demonstrated that our proposal makes the seismic data-inversion process more robust by outperforming the classical approach, especially in the case of uncertainties in the modeled seismic source and in the presence of outliers. When Gaussian noise was considered, the performance of our proposal was very similar to that of the classical approach, although the quantitative indicators of error and similarity showed slight superiority by our proposal (see Table 1).

The results of the simulations show that the seismic inversion based on $\bar{q}$-statistics is a robust methodology for very noisy datasets. In addition, it is worth mentioning that the $\bar{q}$-PSI provides better estimations of model parameters without an additional computational cost compared to the conventional approach. The results also reveal that the optimum $\bar{q}$-value is at the limit $\bar{q}\to 0.01$. In future, we intend to study the sensitivity of our proposed model to multiple reflections (both internal and associated with the free surface), the frequency content of seismic datasets, and the initial model.

We remark that $\bar{q}$-PSI approach for data with outliers provided better results than the conventional PSI approach. Therefore, $\bar{q}$-PSI approach may be a helpful tool in exploration geophysics and a wide variety of other inverse problems for which it is desirable to reduce the heavy-processing of the observed data.

## Figures and Tables

**Table 1:** Main statistics of the PSI results from datasets contaminated by Gaussian noise with different SNRs.

Strategy	*SNR* = 5 dB		*SNR* = 10 dB		*SNR* = 20 dB		*SNR* = 30 dB	
	NRMS	R	NRMS	R	NRMS	R	NRMS	R
Conventional PSI ($\bar{q}\to 1.0$)	0.6144	0.4342	0.3678	0.7587	0.1889	0.9290	0.1019	0.9789
Our proposal ($\bar{q}=0.90$)	0.4784	0.6287	0.2779	0.8528	0.1589	0.9486	0.1004	0.9791
Our proposal ($\bar{q}=0.75$)	0.4662	0.6435	0.2788	0.8520	0.1588	0.9487	0.1003	0.9791
Our proposal ($\bar{q}=0.60$)	0.4068	0.7077	0.2787	0.8520	0.1586	0.9488	0.1003	0.9792
Our proposal ($\bar{q}=0.40$)	0.3594	0.7542	0.2770	0.8536	0.1588	0.9487	0.1004	0.9791
Our proposal ($\bar{q}=0.30$)	0.3162	0.8013	0.2746	0.8549	0.1591	0.9485	0.1004	0.9791
Our proposal ($\bar{q}=0.10$)	0.3176	0.7957	0.2666	0.8623	0.1589	0.9486	0.1003	0.9791
Our proposal ($\bar{q}=0.01$)	0.2947	0.8234	0.2566	0.8698	0.1590	0.9486	0.1004	0.9791

## Data Availability

Not applicable.

## Abbreviations

The following abbreviations are used in this manuscript:

PSI	post-stack inversion
CG	conjugate gradient
NRMS	normalized root mean square
